# Faster ocean warming threatens richest areas of marine biodiversity

**DOI:** 10.1111/gcb.16328

**Published:** 2022-07-14

**Authors:** Stuart C. Brown, Camille Mellin, Jorge García Molinos, Eline D. Lorenzen, Damien A. Fordham

**Affiliations:** ^1^ School of Biological Sciences University of Adelaide Adelaide South Australia Australia; ^2^ Globe Institute, University of Copenhagen Copenhagen Denmark; ^3^ Arctic Research Center Hokkaido University Sapporo Japan; ^4^ Graduate School of Environmental Science Hokkaido University Sapporo Japan

**Keywords:** biogeography, climate analogues, climate change vulnerability, climate stability, climate velocity, conservation management, coral reef, marine biodiversity loss

## Abstract

The vulnerability of marine biodiversity to accelerated rates of climatic change is poorly understood. By developing a new method for identifying extreme oceanic warming events during Earth's most recent deglaciation, and comparing these to 21st century projections, we show that future rates of ocean warming will disproportionately affect the most speciose marine communities, potentially threatening biodiversity in more than 70% of current‐day global hotspots of marine species richness. The persistence of these richest areas of marine biodiversity will require many species to move well beyond the biogeographic realm where they are endemic, at rates of redistribution not previously seen. Our approach for quantifying exposure of biodiversity to past and future rates of oceanic warming provides new context and scalable information for deriving and strengthening conservation actions to safeguard marine biodiversity under climate change.

## Graphical abstract

Using a new approach for quantifying exposure of biodiversity to oceanic warming, we show that future climate change will disproportionately affect Earth's most speciose marine communities, potentially threatening biodiversity in more than 70% of current‐day global hotspots of marine species richness. Near‐term persistence of these richest areas of marine biodiversity will require many species to move well beyond the biogeographic realm where they are endemic, at rates of redistribution not previously seen.

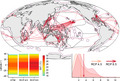

## 1 INTRODUCTION

Virtually every component of Earth's climate system underwent large‐scale change from the end of the Last Glacial Maximum to the early Holocene (approximately 19,000 to 11,000 years ago; Clark et al., 2012), with rates and magnitudes of warming comparable with 21st century projections in many locales and regions (Brown, Wigley, Otto‐Bliesner, Rahbek, & Fordham, 2020; Burke et al., 2018). Whilst not direct analogues of future climatic change due to different forcing (Crowley, 1990), this last deglacial period provides important insights for anticipating the ecological consequences of rapid warming (Fordham et al., 2020; Nolan et al., 2018), including quantifying risks of climate‐driven biodiversity loss (Brown, Wigley, Otto‐Bliesner, Rahbek, et al., 2020), and strengthening conservation management and policy through improved knowledge of biotic responses to climatic stressors (Fordham et al., 2020).

Climatic oscillations in oceanic temperatures during the last deglaciation, and other glacial–interglacial cycles of the late Quaternary (Dansgaard et al., 1993), caused major redistributions of global marine biodiversity (Hewitt, 2000; Pellissier et al., 2014). Many species retreated into localised climatic safe havens during periods of wide‐scale climatic disruption, preventing the extinction of ancient lineages (Pellissier et al., 2014), which promoted differentiation in radiating taxa, resulting in large aggregates of species with restricted ranges (Pinsky et al., 2020). Accordingly, areas where marine species richness is exceptionally high, such as the Coral Triangle in the Indo‐Australian Archipelago, occur in regions where late Quaternary sea surface temperatures (SSTs) have been relatively stable (Pellissier et al., 2014). Thus, maintaining high species richness in past climatic refugia relies, in part, on low‐magnitude rates of future climatic change (Barlow et al., 2018), because resident species are ecologically ill‐equipped to respond rapidly to change (Nguyen et al., 2011; Pellissier et al., 2014; Pinsky et al., 2020).

Recent human‐induced climate change is already affecting Earth's marine biodiversity through shifts in species distributions and abundances (Pecl et al., 2017; Yasuhara et al., 2020), which are causing changes in community structure (Stuart‐Smith et al., 2021; Vergés et al., 2014) that are negatively affecting ecosystem function (Frainer et al., 2017; Kortsch et al., 2015; Pinsky et al., 2020). In the terrestrial realm, biodiverse tropical regions are projected to experience rates of temperature change that exceed even the most abrupt shifts of the late Quaternary, depleting species richness at local and regional scales (Brown, Wigley, Otto‐Bliesner, Rahbek, et al., 2020). However, the prospects for marine species richness in rapidly warming oceans are less clear.

Although exposure of marine biodiversity to absolute changes in past and future oceanic temperatures has been mapped, this has generally been done over short historical time periods (Beaugrand et al., 2015; Burrows et al., 2014; García Molinos et al., 2016), without consideration of the conditions that species evolved in and are adapted to (Hoffmann & Sgrò, 2011). Where a longer‐term perspective has been applied, the exposure of marine biodiversity to large and rapid shifts in oceanic temperature—those operating over decadal‐ to centennial‐scales (Dansgaard et al., 1993)—have been ignored (Beaugrand et al., 2015). In studies where the ecological ramifications of gradual oceanic warming events in the ancient past have been considered, it has been done so only for a single taxonomic group, without directly considering the rate of climatic change as an additional critical driver of change in biodiversity (Yasuhara et al., 2020). Therefore, a better spatiotemporal understanding of patterns of past and future oceanic warming is needed to assess the exposure and, hence, vulnerability (Garcia et al., 2014), of marine biodiversity to future climatic change (Barlow et al., 2018).

Here, we describe and apply a new technique for assessing the exposure of global marine biodiversity (including plants and animals) to human‐induced climatic changes projected for the 21st century. The approach, which builds off methodological advances in modelling the exposure of terrestrial biodiversity to climate change (Brown, Wigley, Otto‐Bliesner, Rahbek, et al., 2020; Trisos et al., 2020), compares spatiotemporal shifts in extreme centennial rates of oceanic warming and absolute SST over the last 21,000 years to future projections, providing a first‐order assessment of the challenges that marine species face this century. Using this new climate change metric, we show that the most biodiverse areas of Earth's oceans are also among the most exposed to accelerated rates of anthropogenic (post‐industrial) climate change and that tracking suitable climatic conditions this century will require species to move distances beyond the biogeographic realms that they evolved in and are endemic to, at rates of movement rarely seen for marine life.

## 2 METHODS

We developed and applied a new protocol for assessing the exposure of marine life to human‐induced climatic change. This was done using data on the distributions of >14,000 species and by calculating two important dimensions of climatic change that affect marine biodiversity: extreme rates of oceanic warming and changes in SST. These climate‐metrics were calculated continuously for the last 21,000 years and for the 21st century using publicly available datasets (Brown, Wigley, Otto‐Bliesner, & Fordham, 2020). Differences in the centennial rate of change in SST during periods of extreme global warming were used to quantify the exposure and, hence, vulnerability of marine biodiversity to climatic change following industrialisation.

### 2.1 Past and future oceanic temperatures

Annual gridded SSTs were extracted from the StableClim database (Brown, Wigley, Otto‐Bliesner, & Fordham, 2020; 2.5° × 2.5° resolution) for the recent past (1850–2005 C.E.), and for current‐day and future (2006–2100 C.E.) climates. Climate data for the ancient past (21,000–100 B.P [1850 C.E.]), was extracted from the TraCE‐21 ka experiment using PaleoView (Fordham et al., 2017). TraCE‐21 ka is a global, coupled ocean–atmosphere‐sea ice‐land surface general circulation model (AOGCM) which simulates the global climate system over the last 21,000 years (Liu et al., 2009). The TraCE‐21 ka simulation has been validated across multiple spatial and temporal scales, where it has been shown to skilfully simulate major climatic events (Liu et al., 2009) and accurately model contemporary climatic conditions (Fordham et al., 2017).

Historic and future projections of SST were based on a multi‐model ensemble average of 19 AOGCMs from the fifth phase of the Coupled Model Intercomparison Project (CMIP5; Taylor et al., 2012). Future forecasts were simulated under two different Representative Concentration Pathways (RCP; Meinshausen et al., 2011; van Vuuren et al., 2011). The multi‐model ensemble data available in StableClim agrees with observed climatological average temperatures at a range of scales (e.g., global *ρ* = .99, root mean square error [RMSE] = 1.98; High tropics [20°S–20°N] *ρ* = .81, RMSE = 1.83). See Brown, Wigley, Otto‐Bliesner, and Fordham et al. (2020) for a full description of the multi‐model ensemble and the RCP scenarios used in this study.

### 2.2 Rapid warming

Periods of rapid global ocean warming were identified and extracted from StableClim using the 95th percentile of “natural” variability of the CMIP5 pre‐industrial control run ensemble (Brown, Wigley, Otto‐Bliesner, & Fordham, 2020). The 95th percentile corresponded to a natural rapid warming rate of 0.18°C/Century. Centuries between 21,000 B.P. and 2100 C.E. with global oceanic warming rates ≥0.18°C/Century were considered extreme and retained for further analysis (Fordham et al., 2019).

Gridded estimates of trend (°C/year) and variability (standard deviation of residuals from the trend; Nadeau et al., 2017) in SST during these extreme centuries of global warming were extracted and averaged for the periods: (i) 21,000 B.P. to 1850 C.E., and (ii) 1850 to 2100 C.E. for both RCP 4.5 and RCP 8.5 scenarios. Spatial estimates of trend were pattern‐scaled using the mean of trends in global mean SST to account for differences in climate forcing (and their effects on global climate trends) between the past and the future (Brown, Wigley, Otto‐Bliesner, Rahbek, et al., 2020). We then calculated the SNR (SNR = |grid cell trend|/grid cell variability) in each grid cell. We used change in SNR as a metric of exposure of biodiversity to extreme rates of centennial climate warming.

### 2.3 Species richness

Marine species richness data came from AquaMaps (Kaschner et al., 2015), which is commonly used for vulnerability assessments of marine biodiversity (Brito‐Morales et al., 2020; García Molinos et al., 2016). Relative probabilities of contemporary species occurrences were based on modelled data and expert knowledge (Kaschner et al., 2015). We discarded distribution data for species modelled with less than 10 occurrence records (i.e., the criterion used by AquaMaps to define data‐scarce species). This resulted in a total of 14,173 marine algae, plant, invertebrate, fish, mammal, and reptile species. A species richness raster was then generated at a 0.5° × 0.5° grid cell resolution by applying an occurrence probability threshold of 0.4 and then summing the total number of species in each grid cell (García Molinos et al., 2016). While the resulting species richness maps generated from AquaMaps are relatively insensitive to this threshold (Jones & Cheung, 2015; Selig et al., 2014), we nonetheless tested the sensitivity of this threshold. To do this we varied the probability threshold between 0 and 0.5 and quantified the overlap between spatial estimates of species richness using the *I* statistic (Warren et al., 2008). We found that all resulting species richness maps were effectively identical at a 0.5° × 0.5° resolution, with values for the *I* statistic ranging from 0.986 to 1.

We matched the spatial resolution of our marine species richness estimates to the spatial resolution of our climate data at a 2.5° × 2.5° grid cell using mean aggregation. We then identified global hotspots of marine species richness as grid‐cells with richness ≥95th percentile of global values, as defined previously (Ramirez et al., 2017). Although we acknowledge that the use of AquaMaps for describing macroecological patterns comes with its own strengths and limitations (Moonlight et al., 2020), we used this data resource to estimate and map species richness and determine the location of biodiversity hotpots because (i) it represents the most comprehensive global dataset of marine species distributions currently available; (ii) the analytical approach underpinning this data repository has been shown to work well, when compared to alternative approaches for generating species range maps at a global scale (Ready et al., 2010); and (iii) high levels of agreement have been shown with other independent estimates of local to regional species richness (Selig et al., 2014). Furthermore, our use of AquaMaps is akin to how IUCN Red List maps have been used elsewhere to determine the exposure of biodiversity to climate change (Trisos et al., 2020).

### 2.4 Exposure of marine biodiversity to future climate change

We used change in grid cell and regional SNR (δ SNR) to quantify the exposure of marine biodiversity to projected rates of oceanic warming following industrialisation. We did this by subtracting past SNR values from post‐industrial to 2100 C.E. values (Brown, Wigley, Otto‐Bliesner, Rahbek, et al., 2020). Here, positive δ SNR values indicate rates of climate change following industrialisation that surpass the most extreme rates of change in the past, whereas negative values indicate rates of climate change that were more rapid in the past.

Biogeographic realms for the world's oceans (Costello et al., 2017) were then intersected with the location of each grid cell. These 30 biogeographic realms are evolutionarily unique areas that harbour large numbers of endemic species (Costello et al., 2017). For grid cells in each realm, we extracted values of past and future SNR. Biogeographic realms with less than three grid cells of area were excluded from any further analysis (*n* = 3). Differences between past and future SNR within realms were assessed using a Kolmogorov–Smirnov test, with *p*‐values adjusted for multiple comparisons following Benjamini and Yekutieli (2001). The proportional overlap (between 0 and 1) in kernel densities of past and future SNR was calculated for each biogeographic realm. The degree of overlap was used as a regional index of exposure to future rapid rates of warming, with low overlap corresponding to high exposure.

For hotspots of marine species richness within each realm, we determined whether centennial rates of warming in SST post‐industrialisation exceeded the 95th percentile of extreme centennial rates of warming that occurred anywhere in the corresponding biogeographic realm prior to industrialisation. We then calculated the percentage of marine biodiversity hotspot cells projected to experience rates of oceanic warming that exceeded these bioregion‐level thresholds (i.e., the regional conditions in which the species persisted from 21,000 B.P. to 1850 C.E.). This approach is conservative compared with competing methods for calculating climate exposure (Williams et al., 2007), because non‐analogue conditions are calculated at the scale of the biogeographic realm (where the majority of species are endemic; Costello et al., 2017) rather than individual grid cells. We tested the sensitivity of projections of climate exposure to the choice of threshold, using thresholds from the 90th to 100th percentile of past (pre‐industrial) SNR. This showed that our calculations of overlap were reasonably stable, with average standard deviation <10% under RCP 4.5 and <3% under RCP 8.5 (range = 0%–25%, dependent on biogeographic realm).

To determine the percentage of global hotspots of marine species richness that are likely to experience no‐analogue rates of 21st century climate warming, we calculated the total number of hotspot grid cells predicted to exceed past extreme SNR values divided by the total number of global hotspot cells. Our approach assumes that species accumulated and evolved in these hotspots of marine species richness over multiple glacial–interglacial cycles (Dynesius & Jansson, 2000; Pellissier et al., 2014). An area‐weighted percentage was also used to determine the average loss of hotspot cells per realm, using the area of the realms as weights.

### 2.5 Climate analogues

Pre‐industrial gridded estimates of annual average sea‐surface temperature were extracted from PaleoView (Fordham et al., 2017), whereas post‐industrialisation estimates were extracted from StableClim for RCP 4.5 and RCP 8.5 (Brown, Wigley, Otto‐Bliesner, & Fordham, 2020). SST data for the pre‐industrialisation period (21,000 BP to 1850 C.E.) was bias corrected using a simple delta bias‐correction (Beyer et al., 2020), calculated using anomalies between the TraCE‐21 data and the StableClim data for the period 1850–1950 C.E. (100–0 B.P.).

To assess the exposure of hotspots of marine species richness to novel climatic conditions, plots of latitudinal mean temperatures (Hovmöller plots) were generated for cells containing species richness ≥95th percentile of global values (see above). Upper grid cell temperatures during the Holocene thermal maximum (HTM)—a variable period between 9000 and 5000 B.P., where tropical ocean temperatures were up to 1°C warmer than pre‐industrial temperatures (Renssen et al., 2012)—were compared with a 21‐year period centred on 2090 (2080–2100 C.E.) under RCP 4.5 and RCP 8.5 and used as an additional measure of exposure to future climate change (García Molinos et al., 2017). We used the VoCC package for R (García Molinos et al., 2019) to calculate the nautical distance (i.e., distances that account for coastal barriers) over which species in marine hotspots would have to disperse to remain within their upper temperature analogue in the future, under both RCPs. For cells where a climate analogue exists in the future, we identified whether they remained in the same or different biogeographic realm and determined the percentage of hotspots that had climate analogues in a different biogeographic realm. We repeated the analysis, restricting the search for climatic analogues to near‐shore Exclusive Economic Zones. We did this to investigate potential future dispersal conditions for species that rely on habitats found close to shore in relatively shallow waters (Kitchel et al., 2022; Nagelkerken et al., 2000).

## 3 RESULTS

**FIGURE 1:**
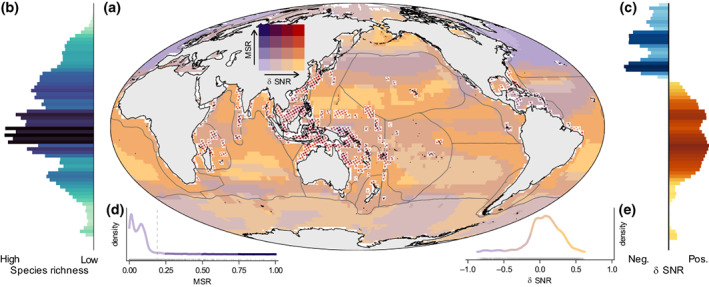
Species richness and rate of oceanic warming. Map (a) shows bivariate relationships between marine species richness (MSR) and pre‐ and post‐industrial (under a future RCP 4.5 scenario) difference in centennial rates of change in sea surface temperature (δ SNR) for periods of rapid oceanic warming. Histograms show latitudinal gradients in MSR (b) and δ SNR (c). Hatched areas in (a) (outlined in white) show hotspots of MSR. Inset plots show density plots of standardised marine species richness (d) and delta SNR (e). Dotted line on (d) corresponds to the 95% threshold for MSR hotspots. Colours on plots (d) and (e) correspond to the colours along the axes of the bivariate plot (a), with changes in colours showing the location of break points. Lines in (a) show the 30 marine biogeographic realms from Costello et al. (2017).

Areas of highest marine species richness occur at tropical and mid‐latitudes, corresponding to regions where SST remained relatively stable during centuries of extreme deglacial oceanic warming at a global scale (Figure 1). However, these most speciose areas of Earth's oceans are projected to experience the greatest relative change in pre‐ and post‐industrial rates of rapid centennial warming by the end of the 21st century (Figure 1), meaning they will be disproportionately exposed to no‐analogue rates of increased SST.

**FIGURE 2:**
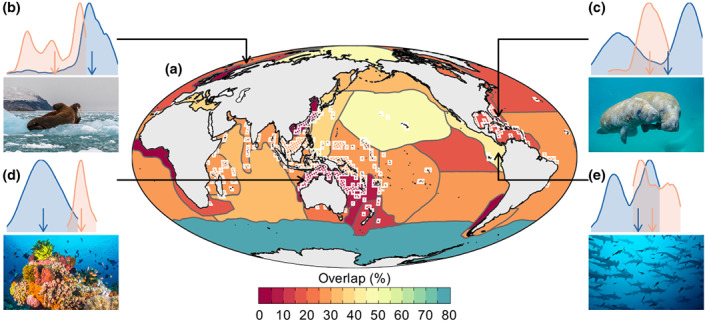
Exposure to future changes in rates of oceanic warming. Map (a) showing overlap (%) in rates of ocean warming in 30 marine biogeographic realms pre‐ and post‐industrialisation (under a future RCP 4.5 scenario). Density plots of rates of rapid warming in four marine biogeographic realms pre‐ and post‐industrialisation: North Atlantic boreal & sub‐Arctic (b), Caribbean and Mexican gulf (c), tropical Australia and Coral Sea (d), and gulf of California (e). Blue indicates pre‐industrialisation, orange indicates post‐industrialisation under RCP 4.5. Coloured arrows in plots (b–d) show median values. Hatched areas in (a) (outlined in white) show hotspots of MSR. Overlap for RCP 8.5 can be found in Figure S2.

At a regional scale, the smallest overlaps in pre‐ and post‐industrial rates of rapid warming are projected to occur in biogeographic realms at tropical and mid‐latitudes (50° South–50° North), based on a comparison of rates of extreme warming using SNR over the last 21,000 years and for the 21st century. Specifically, our modelling indicates that 6 out of 17 tropical and mid‐latitude biogeographic realms will be highly exposed to novel (no‐analogue) rates of future oceanic warming (<10% overlap in pre‐ and post‐industrial rates of extreme warming) by 2100 C.E. under RCP 4.5 (shown in red, Figure 2a). Under RCP 8.5, the number of biogeographic realms in tropical and mid latitudes that will be highly exposed to novel rates of future oceanic warming is projected to increase to 11 (Figure S2).

Although we project significant departures away from the most rapid rates of deglacial oceanic warming by 2100 C.E. for all oceanic biogeographic realms (*p* < .001; Data S1), similar warming rates to those projected for the 21st century occurred more frequently in biogeographic realms at high latitudes (≳50° N). Overlaps in pre‐ and post‐industrial warming rates of up to 50% are projected for biogeographic realms at high latitudes under RCP 4.5 (Figure 2a), increasing to 61% under RCP 8.5 (Figure S2; Data S1). Rates and magnitudes of pre‐industrial centennial warming are projected to have been particularly high in the Offshore and Northwest Atlantic, North American boreal, and Arctic Seas (Figure 2).

At a local scale, we show that locations with plant and animal species richness ≥95th percentile of global values (hatched areas in Figures 1a and 2a) are projected to experience some of the largest shifts in rates of anthropogenic warming. Under RCP 4.5, up to 50% (±11.1% depending on the choice of threshold used to define species ranges, see Section 2) of the total area of these global hotspots of marine species richness will be exposed to rates of climate warming (defined as SNR) this century that exceed even the most abrupt increases in SST in corresponding biogeographic realms over the last ~21,000 years. This figure is projected to rise to up to 81% (±4.7%) under RCP 8.5. The unweighted regional averages were 68% (±8.1%) under RCP 4.5, increasing to 84% (±2.3%) under RCP 8.5.

**FIGURE 3:**
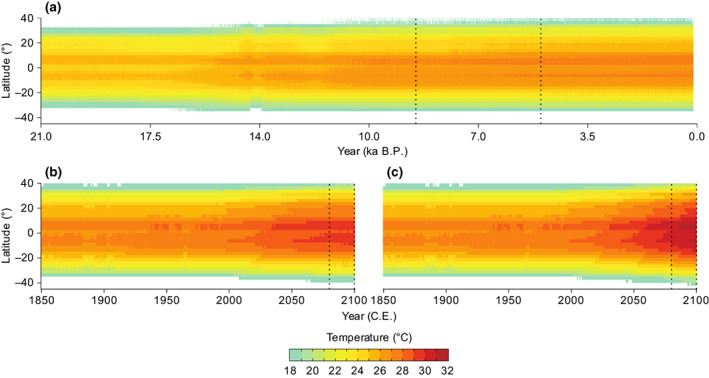
Temporal changes in mean annual sea‐surface temperature for hotspots of marine biodiversity. Hovmöller diagrams showing temperatures for hotspots of marine species richness, grouped by latitudinal bands, for the period 21,000 B.P. to industrialisation (1850 C.E.) (a), and post‐industrialisation to the end of the 21st century under RCP 4.5 (b) and RCP 8.5 (c). Temperatures in (a) have been harmonised with temperatures in (b) using anomalies from a 100‐year period of overlap. Temperatures for RCP 4.5 (b) and 8.5 (c) are based on a multi‐model ensemble average (Brown, Wigley, Otto‐Bliesner, & Fordham, 2020). Black dotted lines in (a) show the time period for the Holocene thermal maximum, and lines in (b) and (c) show the time period where analogous climate conditions from the Holocene thermal maximum were checked.

Impacts of accelerated rates of warming on these global hotspots of marine biodiversity will be amplified by SSTs exceeding absolute temperatures experienced at any time over the last 21,000 years (Figure 3), requiring marine communities in these species‐rich areas to redistribute quickly in order to track their thermal requirements (Burrows et al., 2019). Upper oceanic temperatures experienced during the HTM (the most recent ancient warm period; Renssen et al., 2012) by marine life in hotspots of marine species richness are likely to be on average 1625 km (±922 km) and 2190 km (±997 km) away from their current locations by the end of this century under RCP 4.5 and 8.5, respectively (Figure S3). In 2100, the nearest analogue of upper SST will be in a completely different biogeographic realm to where most species evolved, for 32% and 39% of the richest areas of marine species richness under RCP 4.5 and RCP 8.5, respectively (Figure S4).

**FIGURE 4:**
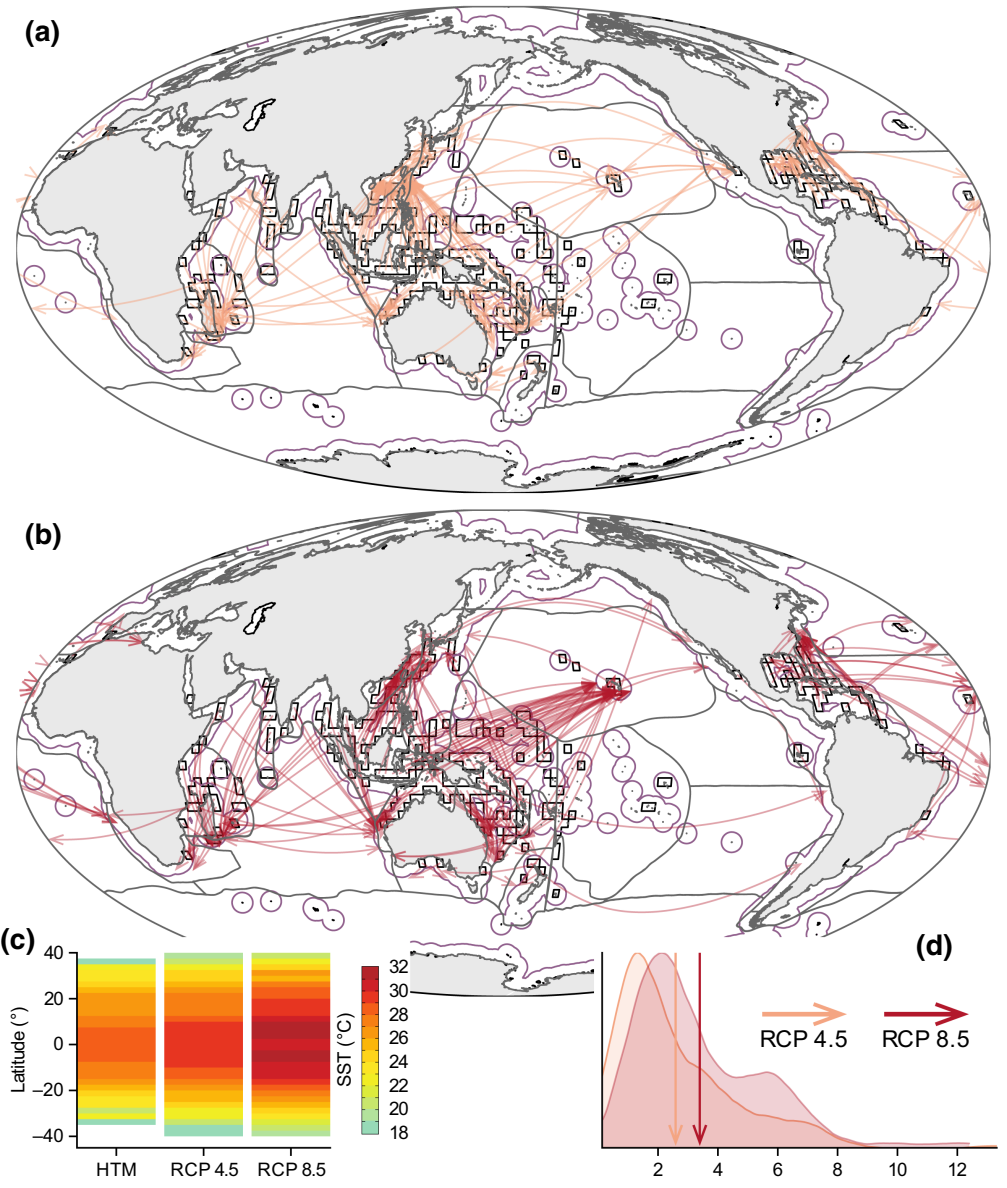
Redistribution of global hotspots of marine species richness under climate change. Maps shows the distance from each biodiversity hotspot cell to the closest near‐shore cell containing an analogue of sea‐surface temperature (SST) under RCP 4.5 (a) and RCP 8.5 (b). The location of analogous cells are restricted to near‐shore locations using the exclusive economic zone (purple outline). Inset (c) shows the latitudinal pattern in analogue conditions between the Holocene thermal maximum to a 21‐year window centred on 2090 C.E. for both RCP 4.5 and RCP 8.5. Inset (d) shows a density plot of the distances (km × 1000) moved under each scenario, with vertical arrows highlighting the mean for RCP 4.5 (orange) and RCP 8.5 (red). Note that nearest analogue distances/locations in (a) and (b) were calculated using nautical distances (i.e., not allowing for dispersal over land) but are plotted using great circles. Gray lines in ocean basins in a‐b show the 30 marine biogeographic realms from Costello et al. (2017). See Section 2 for further details.

Restricting analogue searches to near‐shore locations (<200 nautical miles from the shore; Figure 4), which today provide suitable shallow‐water habitat for locations of exceptionally high species richness (Figure 1), increases further the distance to the nearest SST analogue (RCP 4.5 = 2868 km [±3302]; RCP 8.5 = 4139 km [±3943]; Figure S5). It also increases the proportion of global hotspots of marine species richness (47%–62%) where species must move beyond their biogeographic realm to track their thermal requirements, increasing the need to traverse entire oceanic basins to track thermal requirements.

## 4 DISCUSSION

Our new approach for analysing extreme rates of pre‐ and post‐industrial warming shows that human‐driven climate change will disproportionately affect marine biogeographic realms at tropical and mid‐latitudes this century, exposing 50%–84% (dependent on the warming scenario considered) of the richest areas of global marine biodiversity to future climate‐driven species redistributions. This variation in the spatial pattern of rates of change in SST, acting alone, or in synergy with other human impacts on the marine realm (Gill et al., 2017; Halpern et al., 2008), is likely to cause wide‐scale species extirpations, extinctions, and extensive community reshuffling (Trisos et al., 2020), affecting ecosystem services in areas where governance systems are already struggling to prevent biodiversity losses (Barlow et al., 2018; Oremus et al., 2020).

A higher exposure of marine life to novel oceanic warming by 2100 C.E. in Earth's most specious oceanic regions threatens wide‐scale biodiversity loss (Burrows et al., 2014), not only because the sheer number of species is greater in these areas, but also because ecological communities in these regions are least able to respond to large relative shifts in rates of warming. This is because species in tropical regions—where 93% of hotspots of marine species richness are found, based on area—have small geographic ranges (Pellissier et al., 2014), high ecological specialisation (Foden et al., 2013), limited dispersal capacity (Pinsky et al., 2020), and narrow thermal safety margins (Vinagre et al., 2019), with species living closer to their upper thermal maxima (Nguyen et al., 2011).

The impacts of accelerated rates of warming on these global hotspots of marine species richness are likely to be further amplified by oceanic temperatures exceeding upper absolute temperatures experienced during the HTM, a period in Earth's history (~9000–5000 years ago) when oceanic temperatures in many regions were as warm or warmer than pre‐industrial temperatures (Renssen et al., 2012). However, for many communities of species, tracking these upper thermal requirements will necessitate moving well beyond the biogeographic realm where they are endemic, at rates of redistribution (2020–2090 = ~23–31 km year^−1^) that exceed those observed for most marine taxa (Poloczanska et al., 2013).

Some of the largest shifts in past and future rates of warming are projected to occur in the Coral Triangle and central Indo‐Pacific, which together contain the majority of the world's reef‐building coral species, providing ecosystem services that support the livelihoods of >200 million people (Barlow et al., 2018). Here, and in other global hotspots of marine species richness, conservation interventions are needed immediately to strengthen the ecological and evolutionary resilience of biodiversity to climate change, by improving fisheries management, assisted migration, and the expansion of well‐managed, climate‐smart marine protected areas (Barlow et al., 2018; Brito‐Morales et al., 2020; Oremus et al., 2020). While it has been previously shown that 39% of tropical marine assemblages are projected to have more than 20% of their constituent species exposed to unprecedented temperatures by 2100 based on a post‐industrial baseline (Trisos et al., 2020), further research using a longer‐term baseline could provide important additional information for biodiversity conservation efforts.

In some high‐latitude (≳50° N) biogeographic realms in the Northern Hemisphere, biodiversity has persisted through climatic perturbations during the last deglaciation that approximate those predicted for the future (Figure 2; Figure S2). While these past rapid warming events could have induced selective pressures on organisms, making species in these regions more resilient to rapid rates of future warming (Hoffmann & Sgrò, 2011), absolute temperatures in the Arctic this century are forecast to exceed those experienced over the past 55 million years (Sluijs et al., 2006). These changes are already causing a rapid borealisation of Arctic marine ecosystems (Fossheim et al., 2015; Polyakov et al., 2020) and the redistribution of many Arctic marine species (Frainer et al., 2017), potentially endangering taxa, even those with relatively high dispersal capabilities and low ecological specialisation (Kortsch et al., 2015).

Here we developed and applied a new climate change metric to more than 21,000 years of continuous global climate data to quantify exposure of marine biodiversity to human‐induced oceanic warming, avoiding well‐established limitations in statistical projections of species' range shifts (Yates et al., 2018), and the low availability of demographic, physiological, and evolutionary data for parameterising process‐explicit models of biodiversity change (Pilowsky et al., in press) While climate metrics are highly suited to biodiversity assessments of data‐depauperate and poorly described species (Garcia et al., 2014), which constitutes the majority of marine life (Pacifici et al., 2015), our approach does not distinguish between potentially important inter‐specific differences in adaptive, dispersal, or physiological capacities (Comte & Olden, 2017; Sunday et al., 2012). Therefore, our results should be interpreted somewhat conservatively. Nevertheless, our finding that future ocean warming could cause deleterious biodiversity changes in more than 70% of current‐day global hotspots of marine species richness, provides an important first‐order assessment of the near‐term threat anthropogenic climate change poses to marine biodiversity, particularly in tropical regions. Our analysis did not consider the increasing frequency of short‐term marine heatwaves this century (Oliver et al., 2019), which are likely to further amplify the effects of exposure to accelerated centennial rates of warming, particularly in near‐shore environments (Burrows et al., 2019). Concerningly, abrupt exposure events are projected to occur more frequently in areas of high species richness (Trisos et al., 2020), highlighting the potential for abrupt ecological collapse in global hotspots of marine species richness.

Marine life in tropical regions is ill‐equipped to respond to large relative shifts in rates of future warming and changes in absolute temperatures. Within tropical regions, locations of exceptionally high marine biodiversity will be highly exposed to oceanic warming, making them particularly vulnerable to 21st century climatic change. This new approach and understanding of exposure of marine biodiversity to past and future rates of oceanic warming provides important context and scalable information for deriving and strengthening conservation actions to safeguard marine biodiversity under climate change.

## AUTHOR CONTRIBUTIONS

Stuart C. Brown and Damien A. Fordham conceived and led the project. Jorge García Molinos produced the marine species richness maps. Stuart C. Brown did the analysis and produced all outputs. Stuart C. Brown drafted the manuscript and all authors reviewed and edited the manuscript.

## CONFLICT OF INTEREST

Authors declare that they have no competing interests.

## Supporting information


Figure S1.

Table S1.

## Data Availability

All data and code necessary to reproduce the analysis is available from https://doi.org/10.5281/zenodo.6781192.
