# Supplementary material for: Faster ocean warming threatens richest areas of marine biodiversity
Source: Glob Chang Biol. 2022 Jul 14;28(19):5849–58. doi: 10.1111/gcb.16328 (PMC9544294; doi:10.1111/gcb.16328)
Supplement: Supplementary file 1 — Figure S1. [file GCB-28-5849-s001.pdf]

Faster ocean warming threatens richest areas of marine biodiversity.

Exposure of biodiversity to ocean warming

Stuart C. Brown,[1,2]* Camille Mellin,[1] Jorge García Molinos,[3,4] Eline D. Lorenzen,[2] Damien A. Fordham[1*]

[1]School of Biological Sciences, University of Adelaide, Adelaide, Australia
[2]Globe Institute, University of Copenhagen, Copenhagen, Denmark.
[3]Arctic Research Center, Hokkaido University, Sapporo, Japan
[4]Graduate School of Environmental Science, Hokkaido University, Sapporo, Japan

Email: s.brown@adelaide.edu.au, damien.fordham@adelaide.edu.au

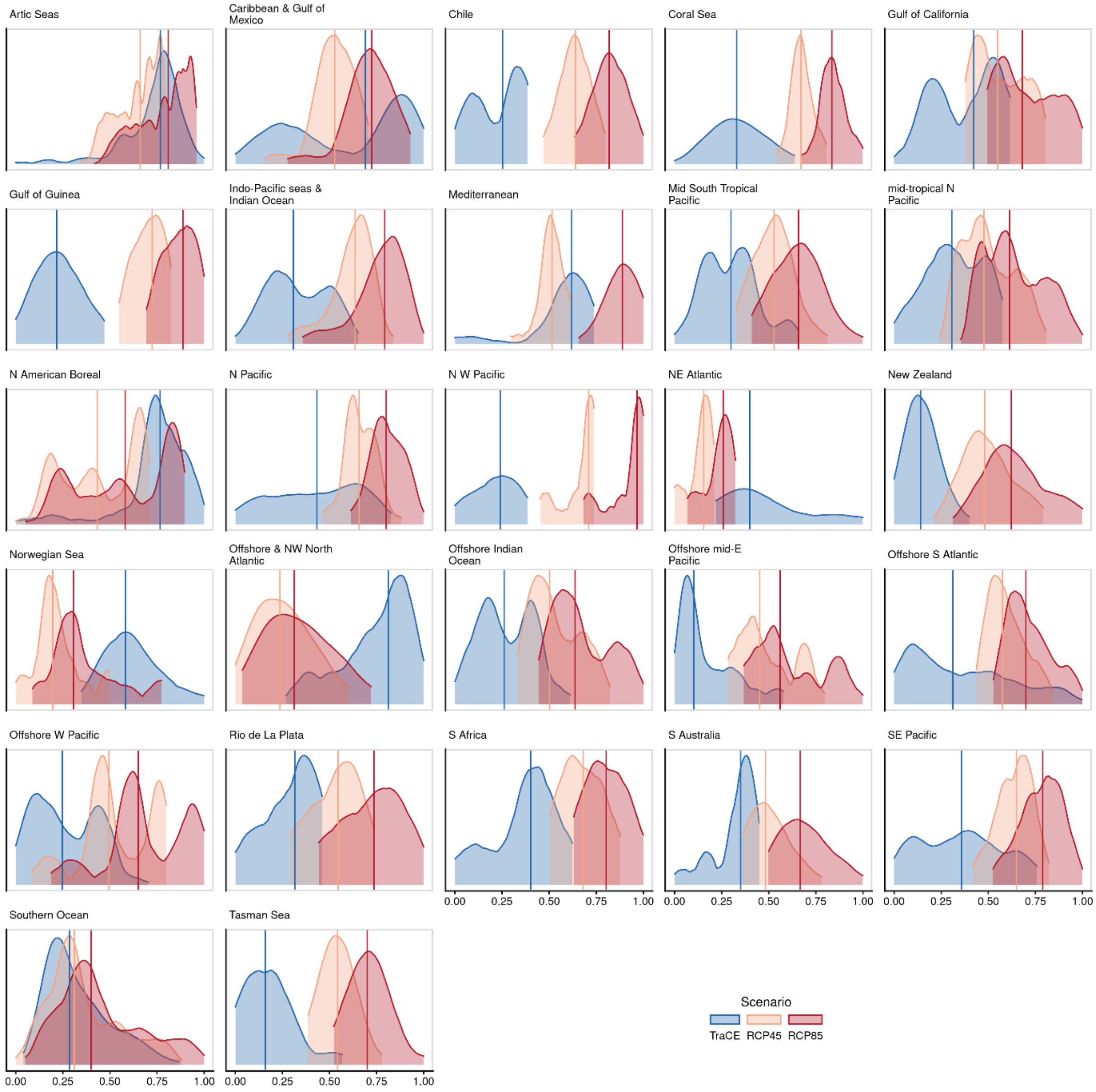

Supplementary Figure 1. Densities of Signal-to-Noise Ratio for sea surface temperature within 27 marine biogeographic realms. Densities are for three climatic scenarios (pre-industrial [blue], RCP 4.5 [orange] RCP 8.5 [red]). Vertical lines represent the median values for the densities. The overlap between the densities, and results of the Kolmogorov–Smirnov test, is provided in Supplementary Table 1. Three realms are excluded from the analysis due to having small sizes (≤ 3 grid cells).

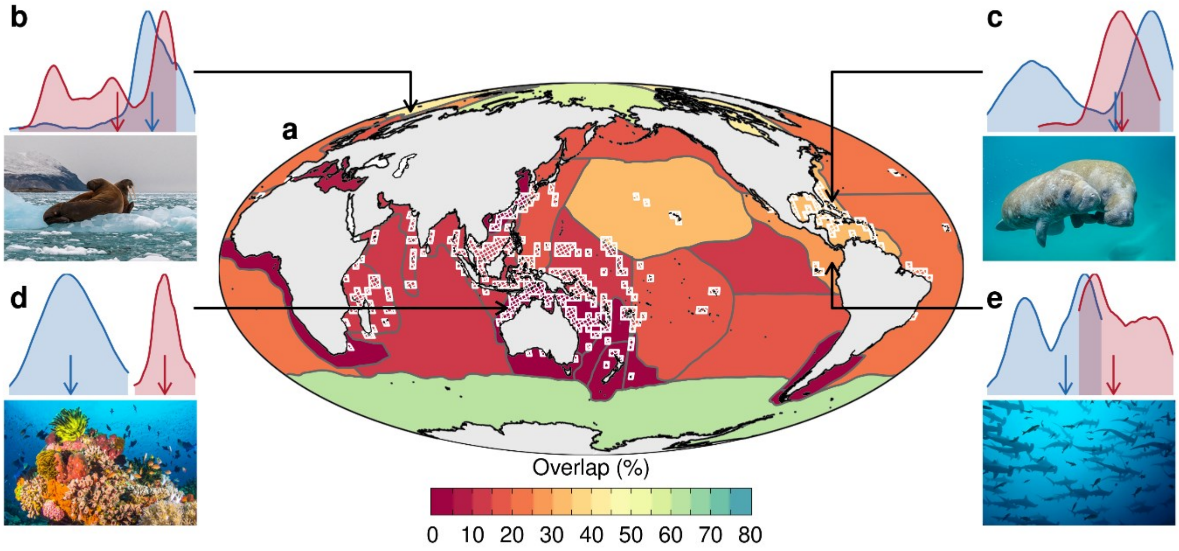

Supplementary Figure 2. Exposure to future changes in rates of oceanic warming under RCP 8.5. Map (**a**) showing overlap (%) in pre- and post-industrial rates of ocean warming in 30 marine biogeographic realms (delineated by the grey lines). Density plots of pre- and post-industrial rates of warming in four marine biogeographic realms: North Atlantic boreal & sub-Arctic (**b**), Caribbean and Mexican Gulf (**c**), Tropical Australia and Coral Sea (**d**), and Gulf of California (**e**). Blue indicates pre-industrial, red indicates post-industrial under RCP 8.5. Arrows in plots show median values. Hatched areas in **a** (outlined in white) show hotspots of marine biodiversity. Overlap for RCP 4.5 can be found in Figure 2.

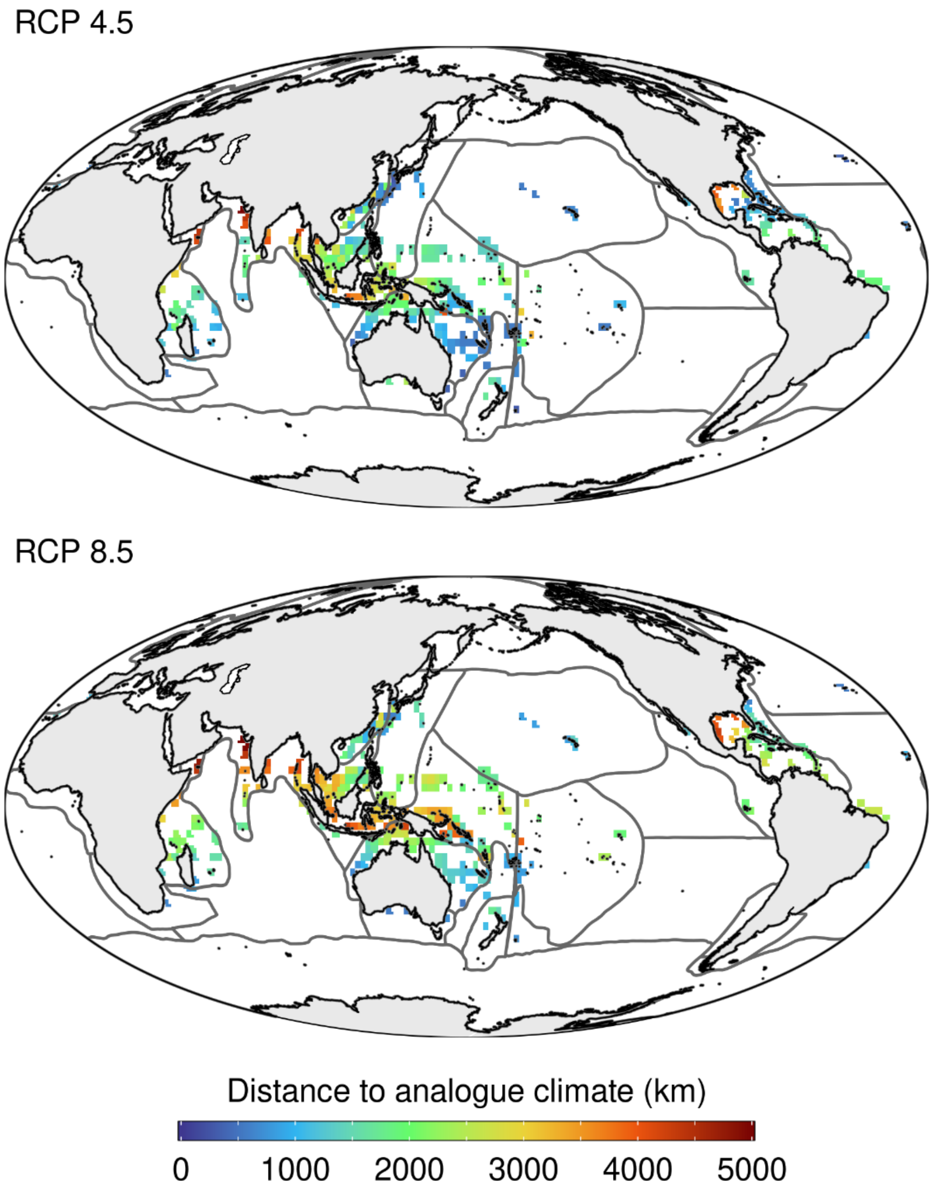

Supplementary Figure 3. Distances to future analogue climates for hotspots of marine biodiversity. Maps show the distances to nearest cell containing an analogue climate under RCP 4.5 and RCP 8.5. Analogue conditions (García Molinos et al., 2017) are from the Holocene thermal maximum (Renssen, Seppä, Crosta, Goosse, & Roche, 2012) (9,000 – 5,000 B.P.), to a 20-year window centered on 2090 C.E. All marine cells were used for analysis (i.e., unrestricted analogue search). Grey lines show the 30 marine biogeographic realms from Costello et al. (2017)

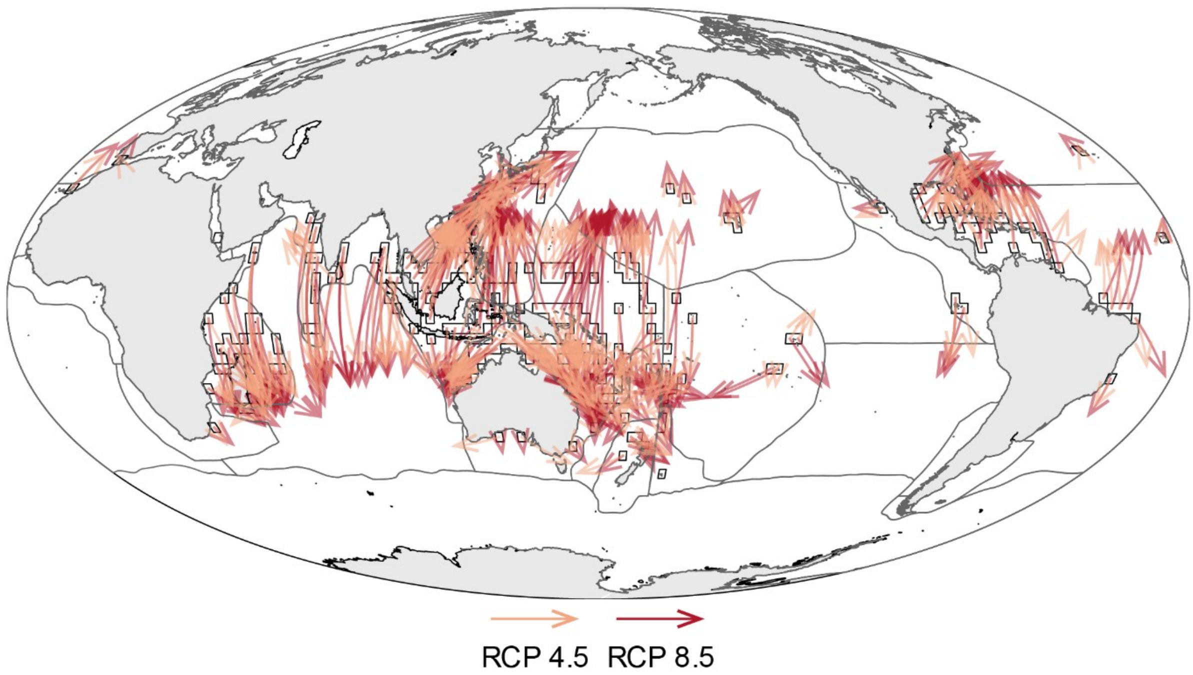

Supplementary Figure 4. Redistribution of marine hotspots under climate change. Map shows the distance from each biodiversity hotspot cell to the closest cell containing an analogue of SST under RCP 4.5 (orange) and RCP 8.5 (red). Analogue conditions (García Molinos et al., 2017) are from the Holocene thermal maximum (Renssen et al., 2012) (9,000 – 5,000 B.P.), to a 20-year window centered on 2090 C.E. Note that nearest analogue distances/locations were calculated using nautical distances (i.e. not allowing for dispersal over land), but are plotted using great circles. All marine cells were used for analysis (i.e., unrestricted analogue search). Grey lines show the 30 marine biogeographic realms from Costello et al. (2017)

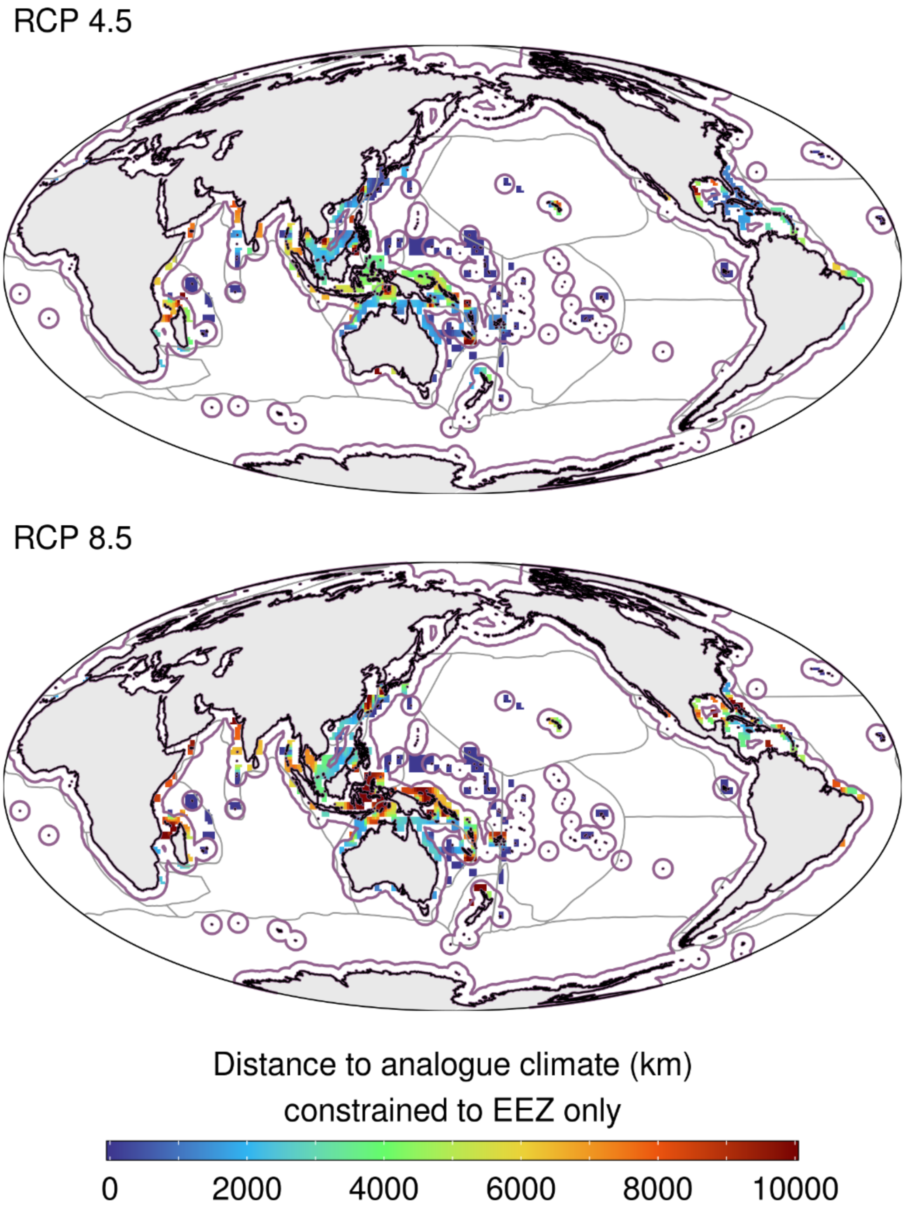

Supplementary Figure 5. Distances to near-shore future analogue climates for hotspots of marine biodiversity. Maps show the distances to nearest near-shore cell containing an analogue climate under RCP 4.5 and RCP 8.5. The location of analogous cells are restricted to near shore locations using the Exclusive Economic Zone (purple outline). Analogue conditions (García Molinos et al., 2017) are from the Holocene thermal maximum (Renssen et al., 2012) (9,000 – 5,000 B.P.), to a 20-year window centered on 2090 C.E. Grey lines show the 30 marine biogeographic realms from Costello et al. (2017)

Supplementary Table 1. (separate file)

Percentage overlap in past and future signal-to-noise ratios (SNR) under both RCP 4.5 and RCP 8.5 within 27 marine biogeographic realms. The results of the Kolmogorov–Smirnov test show whether differences in shape and location were significantly different between past and future SNR. Three realms are excluded from the analysis due to having small sizes (≤ 3 grid cells) - see supporting code. Columns are: Region = marine biogeographic realm; Scenario = representative concentration pathway scenario; Overlap = the amount of overlap between the kernel density estimates; KS = Kolmogorov–Smirnov test statistic; KS_P = Kolmogorov–Smirnov test statistic p-value; KS_S = significance of Kolmogorov–Smirnov test statistic p-value (*** ≤ 0.001, ** ≤ 0.01, * ≤ 0.05); KS_P = Kolmogorov–Smirnov test statistic p-value adjusted for multiple comparisons; KS_Sadj = significance of adjusted Kolmogorov–Smirnov test statistic p-value.

Supplementary References

Costello, M. J., Tsai, P., Wong, P. S., Cheung, A. K. L., Basher, Z., & Chaudhary, C. (2017). Marine biogeographic realms and species endemicity. *Nature Communications, 8*(1), 1057. doi:10.1038/s41467-017-01121-2

García Molinos, J., Takao, S., Kumagai, N. H., Poloczanska, E. S., Burrows, M. T., Fujii, M., & Yamano, H. (2017). Improving the interpretability of climate landscape metrics: An ecological risk analysis of Japan's Marine Protected Areas. *Global Change Biology, 23*(10), 4440-4452. doi:10.1111/gcb.13665

Renssen, H., Seppä, H., Crosta, X., Goosse, H., & Roche, D. M. (2012). Global characterization of the Holocene Thermal Maximum. *Quaternary Science Reviews, 48*, 7-19. doi:10.1016/j.quascirev.2012.05.022
